# Clinical Characteristics and Risk Factors for Disease Severity and Death in Patients With Coronavirus Disease 2019 in Wuhan, China

**DOI:** 10.3389/fmed.2020.00532

**Published:** 2020-08-13

**Authors:** Li Zou, Lijun Dai, Yangyang Zhang, Wenning Fu, Yan Gao, Zhaohui Zhang, Zhentao Zhang

**Affiliations:** ^1^Department of Neurology, Renmin Hospital of Wuhan University, Wuhan, China; ^2^School of Nursing, Tongji Medical College, Huazhong University of Science and Technology, Wuhan, China

**Keywords:** COVID-19, pneumonia, inflammation, lymphopenia, risk factors

## Abstract

**Objective:** To describe the clinical manifestations and outcomes of COVID-19, and explore the risk factors of deterioration and death of the disease.

**Methods:** In this retrospective study, we collected data from 121 COVID-19 cases confirmed by RT-PCR and next-generation sequencing in Renmin Hospital of Wuhan University from January 30, 2019, to March 23, 2020, and conducted statistical analysis.

**Results:** A total of 121 patients were included in our study, the median age was 65 years (IQR, 55.0–71.5 years), and 54.5% cases were men. Among those cases, 52 (43.0%) cases progressed to severe, and 14 (11.6%) died. Overall, the most common manifestations were fever (78.5%) and respiratory symptoms (77.7%), while neurological symptoms were found in only 9.9% of the patients. 70.2% of all the cases had comorbidities, including hypertension (40.5%) and diabetes (20.7%). On admission, cases usually show elevated levels of neutrophils (27.3%), D-dimer (72.6%), Interleukin-6 (35.2%), Interleukin-10 (64.4%), high-sensitivity C-reactive protein (82.6%), and lactate dehydrogenase (62.0%), and decreased levels of lymphocytes (66.9%), CD3 cells (67.2%), and CD4 cells (63.0%). The proportional hazard Cox models showed that the risk factors for severity progression and death included comorbidities (HR: 4.53, 95% CI: 1.78–11.55 and HR: 7.81, 95% CI: 1.02–59.86), leukocytosis (HR: 1.13; 95% CI: 1.05–1.22 and HR: 1.25, 95% CI: 1.10–1.42), neutrophilia (HR: 1.15, 95% CI: 1.07–1.13 and HR: 1.28, 95% CI: 1.13–1.46, and elevated LDH (HR: 1.14, 95% CI: 1.12–1.15 and HR: 1.11, 95% CI: 1.10–1.12). Elevated D-dimer (HR: 1.02, 95% CI: 1.01–1.03), IL-6 (HR: 1.01, 95% CI: 1.00–1.02) and IL-10 levels (HR: 1.04, 95% CI: 1.01–1.07) were also risk factors for the progression of disease severity. Meanwhile, lymphopenia and wake immune responses [e.g., lower CD3, CD4, or CD19 counts (all HR < 1)] were associated with disease deterioration and death.

**Conclusions:** Severe cases and death of COVID-19 are associated with older age, comorbidities, organ dysfunction, lymphopenia, high cytokines, and weak immune responses.

## Introduction

Coronavirus disease 2019 (COVID-19) was first reported in Wuhan, China, and is now spreading in more than 37 countries, including the United States, Japan, South Korea, Australia, and France (1). The pathogen of COVID-19 was identified to be severe acute respiratory syndrome coronavirus 2 (SARS-CoV-2), which is closely related to SARS-like coronavirus (2). In the phylogenetic tree, SARS-CoV-2 was identified as an β-coronavirus 2b lineage. By examining the full-length genome of SARS-CoV-2, it was found that this novel virus shares 87.99% homologous sequences with SARS-like coronavirus (3). Early research has shown that SARS-CoV-2, like SARS-CoV, can infect humans cells expressing angiotensin-converting enzyme 2 (ACE2) (4). Based on the information up to date, SARS-CoV-2 is believed to be the third zoonotic human coronavirus in this century (4). Early reports from Wuhan found that the COVID-19 was SARS-like atypical pneumonia, and can be transmitted from person to person, mainly through respiratory droplets, but also through contact with eye mucosa. Although the virus has been detected in feces, the transmission through the digestive tract remains to be further confirmed. The incubation period is generally 2–14 days. Mild cases are characterized by fever, muscle pain, fatigue, and dry cough. A few patients had neurological and digestive symptoms. In severe cases, dyspnea can occur after a week. Ultimately, the severe patients develop acute respiratory distress syndrome, septic shock, and metabolic acidosis that are difficult to manage (5–7). According to the early epidemiological studies, the basic reproduction number (R0) of SARS-CoV-2 was estimated to be about 2.2 and even more (ranging from 1.4 to 6.5). The low fatality and high morbidity of COVID-19 make it difficult to prevent the spread of the virus (8–10).

Description of clinical features, laboratory indicators, and radiological characteristics of the disease is crucial for early diagnosis of the disease, quarantine of patients, and identification of close contacts. Our goal is to describe the epidemiological, clinical, and laboratory characteristics of patients diagnosed with COVID-19, to compare the clinical characteristics of severe and non-severe cases, and to describe the potential risk factors for disease deterioration and death.

## Methods

### Patients Enrollment and Data Collection

Our study included 121 inpatients diagnosed as COVID-19 who was hospitalized in Renmin Hospital of Wuhan University in China. The diagnosis was based on clinical symptoms, computed tomography (CT), real-time RT-PCR and next-generation sequencing. The admission date was from January 16 to March 3, 2020. All the patients involved in this study lived in Wuhan during the COVID-19 outbreak. We collected data including demographic information (age, gender, and address of usual residence), clinical characteristics (including medical history, comorbidities, symptoms, and signs), initial laboratory findings (hematologic, blood biochemicals, coagulation function, infection-related, and immune-related indices), and clinical outcomes (survival and death). This study was approved by the Hospital Ethics Committee of the Renmin Hospital of Wuhan University [WDRY2020-K136].

According to the diagnostic and treatment guideline for SARS-CoV-2 issued by the Chinese National Health Committee (Version 3-7), the severity of COVID-19 patients was defined. Severe cases were designated when the patients had one of the following criteria: (1) respiratory distress with RR ≥30/min; (2) oxygen saturation ≤ 93% at rest; (3) arterial partial oxygen pressure (PaO_2_)/oxygen absorption concentration (FiO_2_) ≤ 300 mmHg; (4) respiratory failure that needs mechanical ventilation; (5) shock or with organ failure requiring ICU care.

### Procedures

Epidemiological, clinical and outcome data from electronic medical records were reviewed and collected by professionals. Throat swabs samples were collected from all patients and texted by real-time RT-PCR and next-generation sequencing. Laboratory tests on admission included routine blood test [including leucocytes, lymphocytes, eosinophils, hs-CRP, procalcitonin (PCT), serum amyloid A (SAA), and others], coagulation function, serum biochemical tests (including renal and liver function, creatine kinase and LDH), and immunological indicators (including lymphocyte differential counts, inflammatory factors, and SARS-CoV-2-specific antibodies IgM and IgG). These findings are the initial laboratory results of the patient during hospitalization. Thus, these results have not been affected by the treatment.

### Statistical Analysis

Continuous variables were described as medians and interquartile range (IQR), and categorical variables were described as frequency rates and percentages. We used the chi-square (χ^2^) test or the Fisher's exact test to compare categorical data. Mann-Whitney-Wilcoxon test was applied to compare non-normally continuous variables. The proportional hazard Cox models were used to determine HR and 95% CIs between individual factors on progression to severe or death. The sample size varied due to missing data, and missing data were not imputed. The analyses regarding different factors were based on non-missing data. All statistical analyses were performed using SPSS software (V.23.0). Two-tailed *P* values were considered statistically significant at <0.05.

## Results

### Demographics and Clinical Characteristics

**Figure 1:**
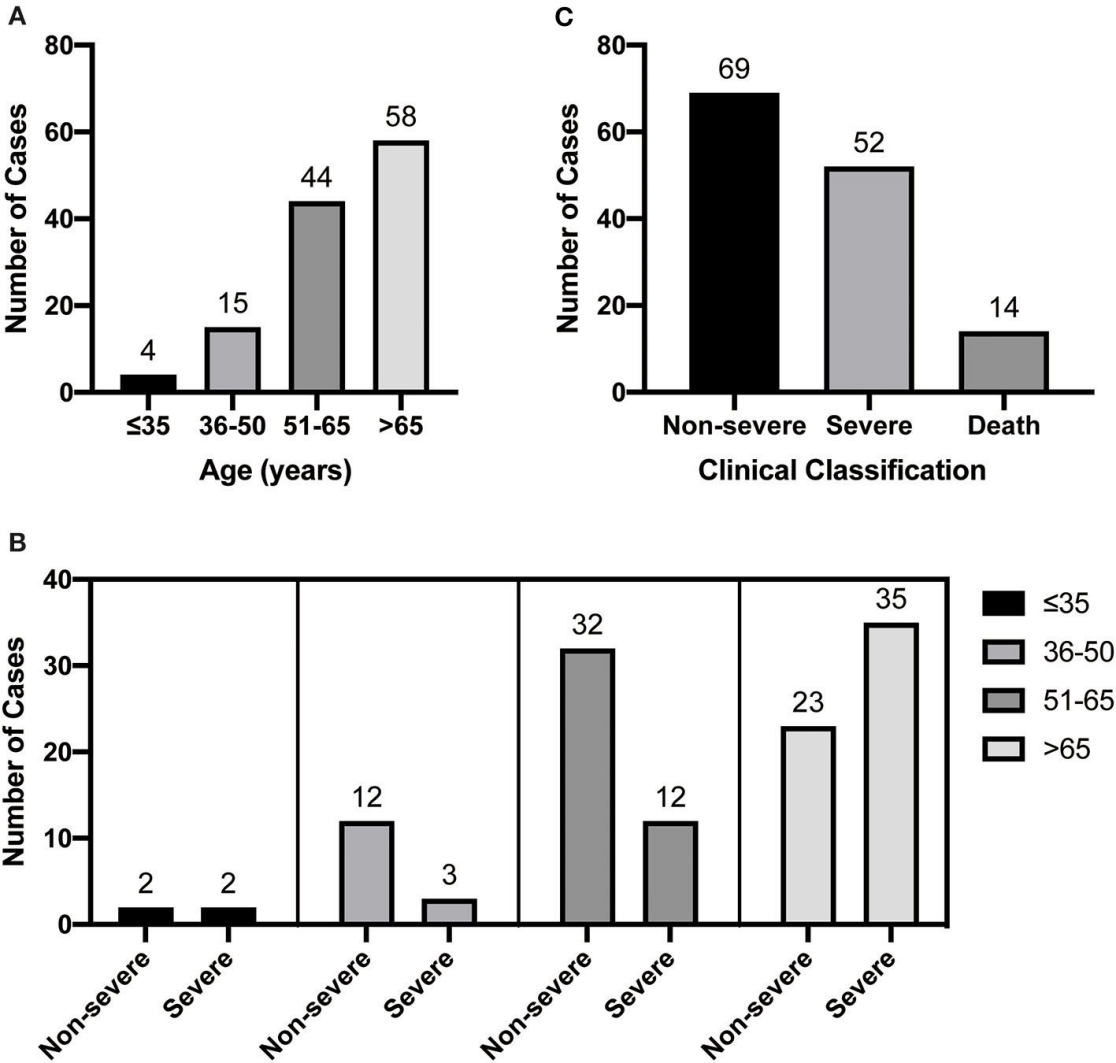
Patient age distribution **(A)**, clinical classification **(B)**, and age distribution of severe and non-serious patients **(C)**.

The demographic and clinical characteristics are shown in Table 1. A total of 121 patients were included in this study. The median age was 65 years (IQR, 55.0–71.5 years) (Figure 1A), of which 54.5% were male. On admission, 69 and 52 patients met the diagnostic criteria for non-severe and severe disease, respectively. The age distribution of these two groups was different (Figure 1C). Of the 121 patients, most (107, 88.4%) survived to discharge, and 14 patients (11.6%) dead (Figure 1B). The most common self-reported symptoms at the time of onset were fever (78.5%) and respiratory symptoms (77.7%), whereas chest distress, fatigue, gastrointestinal symptoms, and neurological symptoms were found in 28.1, 36.4, 23.1, and 9.9% of the patients, respectively. Among the 121 patients, 70.2% had at least one comorbidity, including hypertension, diabetes, cardiovascular and cerebrovascular diseases (excluding hypertension), cancers, and chronic pulmonary diseases. The median age of the severe group was higher than that of the non-severe group (69.5 vs. 60.0, *P* < 0.001). Additionally, the proportion of subjects with at least one comorbidity is more common in the severe group as compared with the non-severe group (88.5 vs. 56.5%, *P* < 0.001). Especially, the incidence of hypertension, cardiovascular and cerebrovascular diseases, and chronic pulmonary diseases in severe cases were significantly higher than that in non-severe cases (all *P* < 0.05). There was no significant difference in demographics and initial clinical characteristics between the survivors and non-survivors.

**Table 1:** Demographic characteristics and initial clinical manifestations of patients with COVID-19 in Wuhan.

**Clinical characteristics and** **symptoms**	**All patients (*n* = 121)**	**Disease severity**		**χ^**2**^**	***P*-value**	**Clinical outcomes**		**χ^**2**^**	***P*-value**
		**Non-severe** **(*n* = 69)**	**Severe** **(*n* = 52)**			**Survival** **(*n* = 107)**	**Death** **(*n* = 14)**		
**Age, Median (IQR)—yrs**	65.0 (55.0–71.5)	60.0 (52.0–68.0)	69.5 (61.5–79.75)		<0.001^a^	64.0 (55.0–70.0)	67.5 (56.75–79.50)		0.229^a^
**Age groups—No., %**									
<65	63 (52.1)	46 (66.7)	17 (32.7)	13.714	<0.001	58 (54.2)	5 (35.7)	1.696	0.193
≥65	58 (47.9)	23 (33.3)	35 (67.3)			49 (45.8)	9 (64.3)		
**Gender—No., %**									
Male	66 (54.5)	34 (49.3)	32 (61.5)	1.799	0.180	57 (53.3)	9 (64.3)	0.606	0.436
Female	55 (45.5)	35 (50.7)	20 (38.5)			50 (46.7)	5 (35.7)		
**Symptoms—No., %**									
Fever on the first admission	95 (78.5)	57 (82.6)	38 (73.1)	1.597	0.206	84 (78.5)	11 (78.6)		1^b^
Chest distress	34 (28.1)	19 (27.5)	15 (28.8)	0.025	0.874	29 (27.1)	5 (35.7)		0.534^b^
Fatigue	44 (36.4)	26 (37.7)	18 (34.6)	0.120	0.729	38 (35.5)	6 (42.9)	0.288	0.591
Respiratory symptoms	94 (77.7)	54 (78.3)	40 (76.9)	0.031	0.861	81 (75.7)	13 (92.9)	2.102	0.147
Gastrointestinal symptoms	28 (23.1)	16 (23.2)	12 (23.1)	<0.001	0.989	25 (23.4)	3 (21.4)		1^b^
Neurological symptoms	12 (9.9)	6 (8.7)	6 (11.5)	0.268	0.605	9 (8.4)	3 (21.4)		0.144^b^
**Coexisting disorders—No., %**									
Any	85 (70.2)	39 (56.5)	52 (88.5)	14.474	<0.001	72 (67.3)	13 (92.9)		0.062^b^
Hypertension	49 (40.5)	22 (31.9)	27 (51.9)	4.942	0.026	40 (37.4)	9 (64.3)	3.781	0.054
Diabetes	25 (20.7)	12 (17.4)	13 (25.0)	1.047	0.306	21 (19.6)	4 (28.6)		0.484^b^
Cardiovascular and cerebrovascular	27 (22.3)	7 (10.1)	20 (38.5)	13.716	<0.001	23 (21.5)	4 (28.6)		0.512^b^
diseases									
Cancer^*^	10 (8.3)	7 (10.1)	3 (5.8)	0.749	0.387	9 (8.4)	1 (7.1)		1^b^
Chronic pulmonary disease^∮^	19 (15.7)	6 (8.7)	13 (25.0)	5.955	0.015	15 (14.0)	4 (28.6)		0.232^b^

*Data are presented as medians (interquartile ranges, IQR) and N (%)*.

*P-value denoted the comparison between different groups*.

a *Mann-Whitney-Wilcoxon was used for continuous variables*.

b *Fisher's exact test*.

* *Cancers referred to any malignancy. All cases were stable disease*.

∮ *Chronic pulmonary disease includes tuberculosis, Chronic obstructive pulmonary disease and Bronchiectasis. All cases were stable and no obvious bacterial infections*.

### Laboratory Findings

Table 2 shows the initial laboratory findings of 121 patients on admission. On admission, 64.0, 27.3, and 18.2% of all patients had lymphopenia, neutrophilia and eosinophilia, respectively. Monocytosis and basophilia were found in 18.2 and 4.1% of all 121 patients, respectively. Some patients showed impaired liver and renal function, as indicated by increased serum aspartate aminotransferase (AST, 6.6%), alanine aminotransferase (ALT, 13.2%), and serum creatinine (sCr, 8.3%), and decreased estimated glomerular filtration rate (eGFR, 39.7%). Most patients showed increased myocardial injury markers. Among 121 cases, 62.0% had elevated serum lactate dehydrogenase (LDH), while only 4.5% had increased creatine kinase muscle-brain isoform (CK-MB). Most patients showed abnormal inflammatory markers. Of 121 patients, 100 had elevated hs-CRP. Meanwhile, 43 of 103 had elevated PCT, and 59 of 96 patients were positive for IgM antibody against SARS-CoV2. Most patients imply a decline of immune function. One hundred and nineteen patients had decreased CD3 (67.2%) and CD4 (67.2%) cell counts. The cell counts of CD3 and CD4 were closely related to the patients' clinical outcomes. In addition, some patients showed abnormal cytokines: 37 of 105 patients (35.2%) showed increased interleukin-6 (IL-6), 56 of 87 patients (64.4%) showed increased interleukin-10 (IL-10), and 19 of 87 patients (21.8%) showed increased tumor necrosis factor (TNF).

**Table 2:** Laboratory Indicators of Patients with COVID-19 in Wuhan.

**Laboratory Tests**	**Reference values**	**No. of patients tested**	**Value, median (IQR)**	**Definition of value deviation from the reference**	**No. of patients with value deviation from reference (%)**
**Hematologic**					
White blood cells, × 10^9^/mL	3.5–9.5	121	5.73 (4.21–8.30)	>10	16 (13.2)
Neutrophils, × 10^9^/mL	1.8–6.3	121	4.06 (2.51–6.56)	>6.5	33 (27.3)
Lymphocytes, × 10^9^/mL	1.1–3.2	121	0.94 (0.68–1.24)	<1.1	81 (66.9)
Monocytes, × 10^9^/mL	0.1–0.6	121	0.44 (0.30–0.57)	>0.65	22 (18.2)
eosinophils, × 10^9^/mL	0.02–0.52	121	0.02 (0.00–0.67)	>0.55	39 (32.2)
basophils, × 10^9^/mL	0–0.06	121	0.02 (0.01–0.03)	>0.065	5 (4.1)
Platelets, × 10^9^/mL	125–350	121	204.0 (161.0–257.5)	<100	12 (9.9)
Hemoglobin, g/L	115–150	121	121.0 (104.0–137.0)	<110	41 (33.9)
**Biochemical**					
ALT, U/L	7–40	121	24.0 (16.0–42.5)	>80	16 (13.2)
AST, U/L	13–35	121	30.0 (20.0–46.5)	>70	8 (6.6)
Albumin, g/L	40–55	121	36.2 (32.65–39.30)	<35	27 (22.3)
Creatinine, μM	41–81	120	65.0 (51.5–78.5)	>150	10 (8.3)
Estimated GFR, mL/min	>90	121	93.86 (78.46–104.90)	<90	48 (39.7)
CK-MB, U/L	40–200	121	63.0 (38.5–96.5)	>200	12 (9.9)
LDH, U/L	120–160	121	296 (210–403)	>250	75 (62.0)
**Coagulation function**					
PT, sec	9–13	118	12.20 (11.40–13.20)	>16	3 (2.5)
APTT, sec	25–31.3	118	28.30 (25.73–30.70)	>42	2 (1.7)
D-dimer, μg/mL	0–0.55	117	1.23 (0.50–4.19)	>0.6	85 (72.6)
FDP, mg/L	0–5	112	4.90 (1.51–13.32)	>6	52 (46.4)
**Infection-related indices**					
hs-CRP, mg/L	0–5	121	NA^*^	>5	100 (82.6)
Procalcitonin, ng/ml	0–2	103	1.19 (0.16–5.40)	>3	43 (41.7)
IgM-NCOV, AU/mL	<10	96	24.03 (9.95–63.02)	>15	59 (62.1)
IgG-NCOV, AU/mL	<10	95	150.87 (113.84–178.05)	>15	3 (3.1)
**Immune-related indices**					
CD3, /μL	723–2737	119	519.0 (359.0–877.0)	<700	80 (67.2)
CD4, /μL	404–1612	119	320.0 (212.0–515.0)	<400	75 (63.0)
CD8, /μL	12–39	119	199.0 (113.0–316.0)	<10	0 (0.0)
CD19, /μL	80–616	118	128.0 (92.0–190.0)	<80	24 (20.3)
CD16+CD56, /μL	84–724	119	107.0 (65.0–189.0)	<80	42 (35.3)
C3, g/L	0.9–1.8	111	1.01 (0.88–1.12)	<0.9	30 (27.0)
C4, g/L	0.1–0.4	111	0.27 (0.20–0.34)	<0.1	1 (0.9)
Interleukin-2, pg/mL	≤ 11.4	87	3.67 (3.28–4.00)	>12	3 (3.4)
Interleukin-4, pg/mL	≤ 12.9	86	3.16 (2.88–3.50)	>12	0 (0.0)
Interleukin-6, pg/mL	≤ 20.0	105	13.54 (6.12–8.00)	>22	37 (35.2)
Interleukin-10, pg/mL	≤ 5.9	87	6.33 (5.41–8.00)	>6	56 (64.4)
Tumor necrosis factor, pg/mL	≤ 5.5	87	3.21 (2.91–4.78)	>6	19 (21.8)
Interferon-γ, pg/mL	≤ 18	87	3.24 (1.95–4.24)	>20	5 (5.7)

*ALT, alanine aminotransferase; AST, aspartate aminotransferase; CK-MB, creatine kinase muscle-brain isoform; LDH, lactate dehydrogenase; PT, prothrombin time; APTT, activated partial thromboplastin time; FDP, fibrinogen degradation products; hs-CRP, high-sensitivity C-reactive protein; GFR, glomerular filtration rate; C3, complement protein C3; C4, complement protein C4; IQR, interquartile range*.

* *The original data of hs-CRP are categorical variables without specific values*.

**Figure 2:**
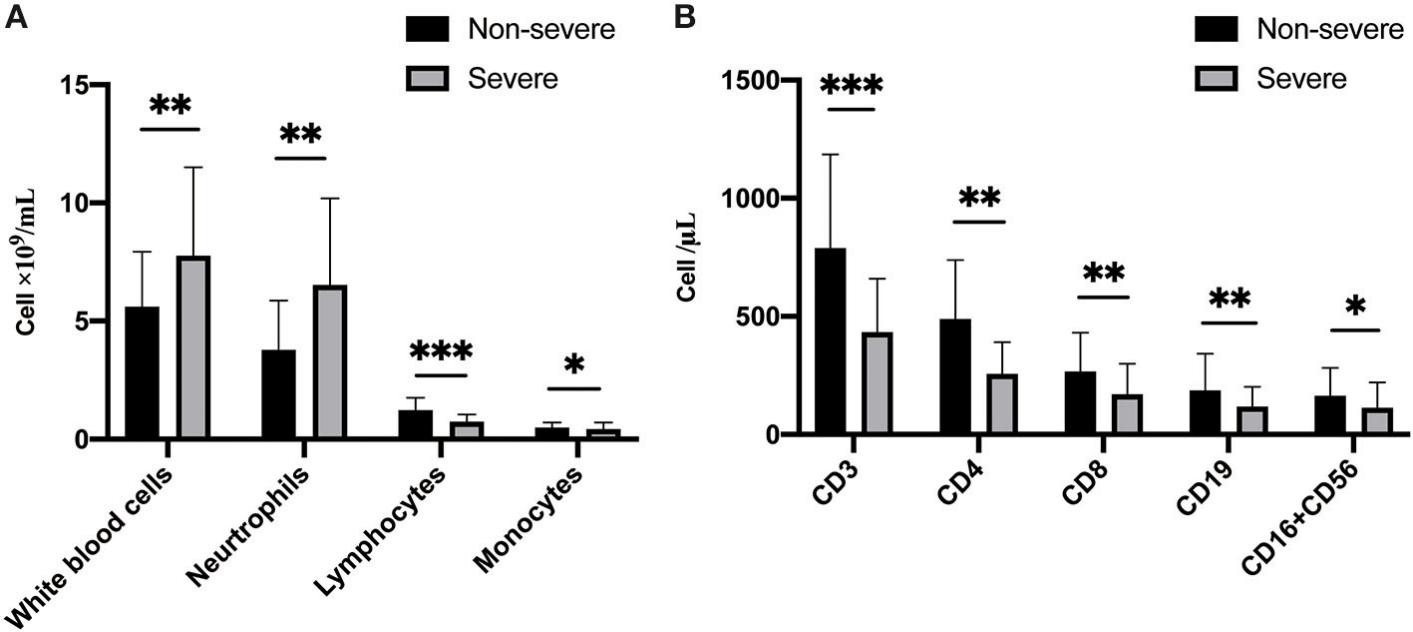
Comparison of immune cells between non-severe and severe patients. **(A)** The total number and types of white blood cells are different between non-severe and severe patients. **(B)** The counts of leukocyte classified by differentiation antigen are different between non-severe and severe patients.

The differences in laboratory findings between non-severe cases and severe cases, as well as survivors and non-survivors are shown in Table 3. Compared with the non-severe group, the severe group showed higher white blood cell (median, 4.83 vs. 7.11, *P* < 0.001) and neutrophil counts (2.90 vs. 5.63, *P* < 0.001) (Figure 2A), as well as higher sCr (59.0 vs. 73.0, *P* < 0.001) and LDH (395.0 vs. 238.0, *P* < 0.001). As for coagulation function, the severe group showed longer PT and APTT, and higher levels of D-dimer and FDP, as well as lower platelet count (all *P* < 0.01) as compared with non-severe group. When compared to the non-severe cases, the severe cases showed abnormal immune function, including fewer CD3, CD4, CD8, CD19, CD16+CD56 T-cells (Figure 2B), and complement C3 levels, as well as higher levels of IL-6 and IL-10 (all *P* < 0.01). Moreover, lower median values of lymphocyte (0.68 vs. 1.05, *P* < 0.001), albumin (34.40 vs. 37.70, *P* < 0.001), and eGFR (86.99 vs. 98.4, *P* < 0.001) were found in severe cases compared to non-severe cases.

**Table 3:** Initial laboratory indices of different disease severity and clinical outcomes.

**Laboratory Tests**	**Reference values**	**Disease severity**		*P*-value^*^	**Clinical outcomes**		*P*-value^*^
		**Non-severe, value, median (IQR)** **(*n* = 69)**	**Severe, value, median (IQR)** **(*n* = 52)**		**Survival, value, median (IQR)** **(*n* = 107)**	**Death, value, median (IQR)** **(*n* = 14)**	
**Hematologic**							
White blood cells, × 10^9^/mL	3.5–9.5	4.83 (4.03–6.70)	7.11 (5.72–10.03)	<0.001	5.46 (4.18–7.68)	7.53 (6.02–6.52)	0.013
Neurtrophils, × 10^9^/mL	1.8–6.3	2.90 (2.37–4.86)	5.63 (4.26–9.19)	<0.001	3.67 (2.44–5.96)	6.52 (4.91–11.97)	0.003
Lymphocytes, × 10^9^/mL	1.1–3.2	1.05 (0.90–1.47)	0.68 (0.52–0.92)	<0.001	0.97 (0.72–1.29)	0.62 (0.32–0.85)	<0.001
Monocytes, × 10^9^/mL	0.1–0.6	0.46 (0.35–0.60)	0.40 (0.26–0.58)	0.049	0.44 (0.32–0.57)	0.31 (0.26–0.58)	0.355
eosinophilis, × 10^9^/mL	0.02–0.52	0.04 (0.00–0.13)	0.01 (0.00–0.075)	0.017	0.03 (0.00–0.11)	0.00 (0.00–0.01)	0.002
basophils, × 10^9^/mL	0–0.06	0.02 (0.01–0.04)	0.02 (0.01–0.03)	0.394	0.02 (0.01–0.03)	0.01 (0.00–0.03)	0.121
Platelets, × 10^9^/mL	125–350	227.0 (186.5–267.0)	172.0 (112.5–234.0)	<0.001	218.00 (175.00–259.00)	123.00 (89.00–174.25)	<0.001
Hemoglobin, g/L	115–150	120.0 (109.0–131.0)	122.0 (89.5–141.0)	0.838	121.00 (105.00–134.00)	132.00 (91.50–145.00)	0.612
**Biochemical**							
ALT, U/L	7–40	24.0 (16.5–45.5)	24.0 (13.5–41.0)	0.444	24.00 (16.00–43.00)	21 (13.50–40.50)	0.53
AST, U/L	13–35	26.0 (19.0–45.0)	35.0 (21.0–53.5)	0.15	29.00 (20.00–47.00)	33.50 (24.25–44.50)	0.381
Albumin, g/L	40–55	37.7 (33.75–40.8)	34.40 (31.30–37.25)	0.001	36.20 (32.50–39.40)	35.70 (32.68–38.13)	0.691
Creatinine, μM	41–81	59.0 (49.0–72.0)	73.0 (54.5–83.0)	0.003	65.00 (51.00–78.00)	70.50 (52.75–85.00)	0.418
Estimated GFR, mL/min	>90	98.4 (89.5–107.5)	86.99 (66.83–97.39)	<0.001	94.78 (80.30–105.00)	90.81 (71.15–102.30)	409
CK-MB, U/L	40–200	55.0 (35.0–87.5)	70.0 (42.5–116.5)	0.108	59.00 (38.00–89.00)	69.00 (34.25–326.50)	0.345
LDH, U/L	120–160	238.0 (189.0–321.0)	395.0 (274.5–589.0)	<0.001	286.00 (207.00–381.00)	412.50 (248.75–648.25)	0.013
**Coagulation function**							
PT, sec	9–13	11.80 (11.02–12.58)	13.00 (12.10–13.80)	<0.001	12.00 (11.30–13.03)	13.45 (12.70–14.18)	0.002
APTT, sec	25–31.3	27.10 (25.23–29.63)	29.30 (27.50–31.80)	0.001	27.90 (25.48–30.53)	28.70 (27.55–32.83)	0.203
D-dimer, μg/mL	0–0.55	0.85 (0.41–1.76)	3.93 (1.23–13.01)	<0.001	1.21 (0.49–13.15)	2.91 (1.03–10.88)	0.081
FDP, mg/L	0–5	2.63 (0.89–5.97)	13.03 (6.41–67.48)	<0.001	4.54 (1.48–13.15)	8.63 (4.57–90.28)	0.026
**Immune-related indices**							
CD3, /μL	723–2737	755.0 (487.0–1055.0)	394.50 (287.25–588.0)	<0.001	536.00 (403.00–899.50)	284.50 (133.25–483.75)	<0.001
CD4, /μL	404–1612	450.0 (301.0–649.0)	240.50 (165.00–323.75)	<0.001	356.00 (240.50–561.00)	140.50 (75.25–247.75)	<0.001
CD8, /μL	12–39	221.0 (133.0–348.0)	146.00 (74.00–253.75)	<0.001	203.00 (120.00–325.00)	144.00 (40.25–242.75)	0.049
CD19, /μL	80–616	155.0 (114.0–202.0)	108.50 (57.00–145.75)	<0.001	138.00 (101.00–192.50)	58.00 (37.75–136.60)	0.004
CD16+CD56, /μL	84–724	130.0 (79.0–216.0)	85.50 (44.00–111.75)	<0.001	11.00 (67.00–197.50)	76.00 (38.00–107.75)	0.037
C3, g/L	0.9–1.8	1.07 (0.93–1.17)	0.93 (0.84–1.06)	0.003	1.03 (0.91–1.15)	0.86 (0.80–0.98)	0.011
C4, g/L	0.1–0.4	0.27 (0.20–0.34)	0.25 (0.20–0.33)	0.46	0.27 (0.21–0.34)	0.19 (0.16–0.27)	0.011
Interleukin-6, pg/mL	≤ 20.0	7.70 (5.27–19.71)	28.91 (15.89–57.70)	<0.001	Null value		–
Interleukin-10, pg/mL	≤ 5.9	6.11 (5.10–7.07)	7.75 (6.15–9.37)	0.006	Null value		–
Interferon-γ, pg/mL	≤ 18	3.18 (2.83–3.72)	3.63 (3.07–4.88)	0.01	Null value		–

*ALT, alanine aminotransferase; AST, aspartate aminotransferase; CK-MB, creatine kinase muscle-brain isoform; LDH, lactate dehydrogenase; PT, prothrombin time; APTT, activated partial thromboplastin time; FDP, fibrinogen degradation products; hs-CRP, high-sensitivity C-reactive protein; IL-6, interleukin-6; IQR, interquartile range*.

* *P-value was determined by Mann-Whitney-Wilcoxon*.

### Clinical Outcomes

The differences of laboratory indicators between the survivors and non-survivors are shown in Table 3. The non-survivors showed higher leucocyte and neutrophil counts, higher levels of LDH and FDP, longer PT, and fewer counts of lymphocytes, platelets, CD3, CD4, CD8, CD19 T-cells, and lower levels of complement C3 and C4 as compared to the survivors (all *P* < 0.05).

Table 4 shows the bivariate cox regression of factors associated with disease progression from onset to severe disease or to death. Patients over 65 were at higher risk of developing severe disease than those under 65 (HR, 2.26; 95% CI, 1.23–4.05; *P* < 0.01). Cases with comorbidities were 4.53 times more likely to progress to severe than cases without comorbidities (HR, 4.53; 95% CI, 1.78–11.55, *P* < 0.01). In addition, Cox regression models illustrated that comorbidities (HR, 7.81; 95% CI, 1.02–59.86; *P* < 0.05), leukocytosis (HR, 1.25; 95% CI, 1.10–1.42; *P* < 0.01), and neutrophilia (HR, 1.28; 95% CI 1.13–1.46; *P* < 0.001) were risks factors for mortality. Elevated LDH was also a risk factor for the severity deterioration and the development of death (HR, 1.14; 95% CI 1.12–1.15; *P* < 0.001 and HR, 1.11; 95% CI 1.10–1.12; *P* < 0.001). Fewer platelets, CD3 or CD4 counts, lower complement C3 level, lymphopenia, and elevated APTT, IL-6 or IL-10 also were related to the progression from oneset to death (all *P* < 0.05).

**Table 4:** Bivariate Cox regression of factors associated with disease progression from onset to severe disease or to death.

**Patient characteristics and findings**	**Severe**		**Death**	
	**HR (95% CI)**	***P*-value**	**HR (95% CI)**	***P*-value**
**Clinical characteristics**				
Age (≥65 vs. <65), y	2.26 (1.23–4.05)	0.006	2.06 (0.69–6.17)	0.195
**Comorbidities**				
Cardiovascular and cerebrovascular diseases (yes vs. no)	2.54 (1.41–4.58)	0.002	2.79 (0.80–9.71)	0.106
Any (yes vs. no)	4.53 (1.78–11.55)	0.002	7.81 (1.02–59.86)	0.048
**Laboratory findings**				
White blood cells, × 10^9^/mL	1.13 (1.05–1.22)	0.001	1.25 (1.10–1.42)	0.001
Neurtrophils, × 10^9^/mL	1.15 (1.07–1.23)	<0.001	1.28 (1.13–1.46)	<0.001
Lymphocytes, × 10^9^/mL	0.16 (0.07–0.39)	<0.001	0.12 (0.03–0.35)	<0.001
Platelets, × 10^9^/mL	0.89 (0.86–0.92)	<0.001	0.88 (0.85–0.91)	<0.001
LDH, U/L	1.14 (1.12–1.15)	<0.001	1.11 (1.10–1.12)	<0.001
APTT, sec	1.05 (1.01–1.09)	0.027	1.04 (0.95–1.14)	0.35
D-dimer, μg/mL	1.02 (1.01–1.03)	<0.001	1.02 (0.99–1.04)	0.155
CD3, /μL	0.87 (0.83–0.91)	<0.001	0.85 (0.81–0.89)	<0.001
CD4, /μL	0.83 (0.79–0.89)	<0.001	0.82 (0.78–0.88)	<0.001
CD19, /μL	0.86 (0.82–0.90)	0.061	0.83 (0.79–0.87)	0.035
C3, g/L	0.39 (0.073–2.05)	0.264	0.26 (0.03–1.37)	<0.001
Interleukin-6, pg/mL	1.02 (1.01–1.03)	0.033	NA	
Interleukin-10, pg/mL	1.04 (1.01–1.07)	0.013	NA	

*LDH, lactate dehydrogenase; APTT, activated partial thromboplastin time; FDP, fibrinogen degradation products; NA, not available*.

## Discussion

This retrospective descriptive study included 121 confirmed cases of COVID-19 with a median age of 65. The median age of the severe group was 9.5 years older than that of the non-severe group. The proportion of males in the severe group was as high as 61.5%, compared with 49.3% in the non-severe group. Moreover, according to the bivariate Cox regression analysis, the risk of adverse outcomes in patients with age over 65 years was 2.28 times that of patients under 65 years of age. Fever and respiratory symptoms were the dominant initial symptoms while neurological symptoms and gastrointestinal symptoms are not as common as respiratory symptoms, indicating the difference in viral tropism as compared with influenza. This result is consistent with other reports (11, 12).

In addition, there is no obvious difference in the initial clinical manifestations between non-severe and severe cases, or survivors and non-survivors, suggesting that the progression of the disease cannot be predicted only based on the symptoms on admission. This conclusion was also confirmed by the proportional hazard Cox models (data not shown). In our study, 70.2% of all cases had at least one comorbidity, including hypertension, diabetes, cardiovascular and cerebrovascular diseases, cancers, or chronic pulmonary diseases. Likewise, the adopted Cox models showed that cardiovascular and cerebrovascular diseases were the vital risk factors to predict the severity of the disease.

Analysis of the laboratory indicators shows that the increase of leukocytes, neutrophils, D-dimer, LDH, IL-6, IL-10, TNF, and IFN-γ, and the decrease of lymphocytes, CD3, CD4, and platelets counts were related to disease progression and adverse clinical outcomes. Interestingly, we found eosinophilia in 18.2% of the patients, which seems to be indicator of better outcomes. This observation is different from an early study (13). The eosinophil counts in non-severe cases are significantly higher than that in severe cases. When compare the survivors and non-survivors, the former also show higher eosinophil counts. All cases did not have history of allergic diseases such as asthma. It is conceivable that inflammatory factors released by the eosinophils can trigger cough, sputum discharge, and sneezing, which are beneficial for the patients to expel the virus and fight against viral toxicity. The exact role of eosinophils in COVID-19 remains to be further illustrated.

In our study, leukocytosis was often accompanied by neutropenia and lymphopenia, and this phenomenon was more common in severe cases. Consistently, the median value of neutrophils was higher in the severe group than in the non-severe group, which could be a sign of excessive inflammatory response. According to the bivariate Cox regression analysis, neutropenia was a risk factor for severe cases and adverse clinical outcomes. Lymphocytosis has been reported by many clinical investigators and is also common in severe cases. We also found that CD3, CD4, CD19, and CD16 + CD56 T-cell counts decreased to varying degrees and were more pronounced in severe cases and deaths. Significantly reduced lymphocyte counts in the severe cases indicate impaired immune function in these patients.

The pathological and anatomical examination of patients with COVID-19 also showed that the number of CD4 and CD8 T-cells was greatly reduced (14). Therefore, we speculate that impaired immune function is one of the risk factors for elderly patients with poor clinical outcomes. Although recent studies have emphasized that SARS-CoV-2 can infect immune cells through CD147, thereby affecting the immune function, the cause of immune function impairment in patients with COVID-19 needs to be further studied (15).

Another notable finding was that IL-6, IL-10, TNF, and IFN-γ were generally elevated in severe cases. Studies showed the that inflammatory factor storm was one of the crucial causes of disease progression and adverse clinical outcomes (16–18). Therefore, the prevention of inflammatory factor storm should be considered for patients with severe COVID-19 (19, 20).

Many subjects show increased serum CK-MB and LDH, indicating the SARS-CoV-2 can cause myocardial damage. This coincides with the latest COVID-19 research findings of the Department of Cardiology and Cardiac Ultrasound (21, 22). Furthermore, the bivariate Cox regression analysis emphasized higher LDH as a risk factor for the development of severity disease and the progression of death. It is worth noting that myocardial damage should be treated properly to reduce mortality.

Coagulation dysfunction was another risk factor for death. There are also reports of vascular embolism in patients with COVID-19 (23, 24). In this study, 72.6% and 46.4% of the cases showed high D-dimer and FDP, respectively. Compared with non-severe cases (median, 3.93), the D-dimer of severe cases (median, 0.85) is significantly increased, suggesting that the increase of D-dimer may be one of the risk factors for the deterioration of the disease, and attention should be paid to monitor coagulation function. Longer PT and APTT, with higher D-dimer and FDP in the initial laboratory findings of dead cases may suggest the possibility of disseminated intravascular coagulation (DIC).

The strengths of the present study as follows: The inpatients of this research came from Wuhan, where is the hit-hardest region of China in this epidemic. Understanding the clinical manifestations and prognosis of cases, and analyzing the risk factors of disease progression and death can provide a theoretical basis for clinical diagnosis and intervention. Clinicians, especially critical care physicians, have a deep understanding of the condition of COVID patients upon admission, which is more helpful in controlling the progression of the disease and improving the prognosis. Limitations of this study were that this study conducted in a single hospital with limited sample size. Information and selection bias may influence the result. A larger-scale multicenter study of COVID-19 patients will help to further determine the clinical features and risk factors of this disease.

## Conclusion

This study included 121 confirmed COVID-19 cases with a median age of 65. Fever and respiratory symptoms were the most common manifestations. Age and comorbidity were the risk factors for death and disease progression. Myocardial injury markers and renal function also suggest the possibility of deterioration. Abnormal coagulation function suggests the risk of DIC in severe cases. The immune-related laboratory results indicate the presence of inflammatory factor storm and impaired immune function, which are closely related to the adverse clinical outcomes. Thus, these indicators deserved special attention. Early intervention should be considered to treat impaired immune functions to reduce mortality. Most factors that are related to the development of disease deterioration were associated with death, indicating the same pathophysiological mechanisms mediate the progression from hospital admission to severity worsening and from onset to death.

## Data Availability Statement

All datasets presented in this study are included in the article/supplementary material.

## Ethics Statement

This study was approved by the Hospital Ethics Committee of the Renmin Hospital of Wuhan University [WDRY2020-K136]. Written informed consent for participation was not required for this study in accordance with the national legislation and the institutional requirements.

## Author Contributions

LZ had full access to all of the data in the study and take responsibility for the integrity of the data and the accuracy of the data analysis. WF were helped statistical analysis and gave technical support. LD and LZ draft the manuscript. LZ and YZ designed the study. YZ and YG helped in the acquisition of data. ZheZ and ZhaZ were responsible for data interpretation and the critical revision of the manuscript. All authors contributed to the article and approved the submitted version.

## Conflict of Interest

The authors declare that the research was conducted in the absence of any commercial or financial relationships that could be construed as a potential conflict of interest. The reviewer XW declared a shared affiliation with one of the reviewers WF to the handling editor at time of review.
